# Operationalisierung der Bestimmung des Barthel-Index mittels „Barthel plus“

**DOI:** 10.1007/s00391-025-02436-2

**Published:** 2025-03-31

**Authors:** Sonja Krupp, Jennifer Kasper, Christina Gollmer, Friedrich Balck, Meike Kasten, Martin Willkomm

**Affiliations:** 1 Forschungsgruppe Geriatrie Lübeck, Krankenhaus Rotes Kreuz Lübeck Geriatriezentrum, Marlistr. 10, 23566 Lübeck, Deutschland; 2 Klinik für Psychiatrie und Psychotherapie, Universität zu Lübeck, Ratzeburger Allee 160, 23562 Lübeck, Deutschland; 3 Psychosoziale Medizin und Entwicklungsneurowissenschaften, Universitätskrankenhaus Carl Gustav Carus, Fetscherstr. 74, 01307 Dresden, Deutschland

**Keywords:** Selbstversorgung, Pflegebedürftigkeit, Assessment, Prävention, Geriatrie, Self-care, Care dependency, Assessment, Prevention, Geriatrics

## Abstract

**Hintergrund:**

Der Barthel-Index ist das gebräuchlichste Instrument zur Einschätzung der basalen Selbstversorgungsfähigkeit. Er erfasst Beeinträchtigungen jedoch erst im Stadium personellen Unterstützungsbedarfs.

**Ziel der Arbeit:**

Von der Langfassung des Hamburger Einstufungsmanuals (HEMB-L) ausgehend wurde in Rücksprache mit Pflegefachpersonal der Barthel plus (Bplus) als Operationalisierung entwickelt; diese bildet über eine punktneutrale Subskalierung auch kompensierte Beeinträchtigungen ab. Der Artikel stellt das Instrument und seine Güteeigenschaften vor.

**Material und Methoden:**

Bei Patienten in der Akutgeriatrie wurde der Bplus 2‑mal im Abstand von über einer Woche eingesetzt; dabei wurden Audioaufnahmen gemacht. Einen, 12, 24 und 36 Monate nach der Entlassung wurde der Bplus telefonisch erhoben. Für die Änderungssensitivität wurde die Effektstärke (Cohens d) während des stationären Verlaufs errechnet. Die Interrater-Reliabilität und Übereinstimmung mit dem HEMB‑L wurden unter Einbezug der Bewertung der Audiodateien durch verblindete Untersucher bestimmt.

**Ergebnisse:**

Zwischen 29.04.2019 und 25.06.2021 wurden 124 Patienten in die Studie eingeschlossen. Die anhand des Bplus und HEMB‑L erhobenen Werte für den Barthel-Index unterschieden sich nicht signifikant. Die Interrater-Reliabilität, interne Konsistenz und Änderungssensitivität des Bplus waren mit jeweils > 0,9 hoch. Bei selbstständig, jedoch unter Beeinträchtigung ausgeübten Tätigkeiten bestand ein erhöhtes Risiko für den Eintritt von Pflegeabhängigkeit im Verlauf.

**Diskussion:**

Der Bplus kann unter Wahrung des Summen-Scores im Vergleich zum HEMB‑L von Verlust der Selbstständigkeit bedrohte Fähigkeiten markieren und so die Prävention fortschreitender Pflegebedürftigkeit erleichtern.

## Abstract (en)

**Background:**

The Barthel Index is the most commonly used instrument for assessing basic self-care ability; however, it only records impairments first at the stage of needing personal support.

**Objective:**

Based on the long version of the Hamburg Classification Manual (HEMB-L), the Barthel plus (Bplus) was developed in consultation with specialist nursing personnel as an operationalization that also depicts compensated impairments via point-neutral subscaling. The article presents the instrument and its quality characteristics.

**Material and methods:**

For patients in acute geriatric care the Bplus was employed twice at an interval of more than 1 week and audio recordings were made. The Bplus was assessed by telephone 1, 12, 24 and 36 months after discharge. The effect size (Cohen’s d) was calculated for the sensitivity to change during the inpatient course. The interrater reliability and agreement with the HEMB‑L were determined taking the evaluation of the audio files by blinded examiners into account.

**Results:**

Between 29 April 2019 and 25 June 2021 a total of 124 patients were included in the study. The Barthel Index scores obtained using the Bplus and HEMB‑L did not show a significant difference. The interrater reliability, internal consistency and change sensitivity of the Bplus were high, each at > 0.9. For activities performed independently but with impairment, there was an increased risk of care dependency occurring over the course of the study.

**Conclusion:**

While maintaining the same sum score as the HEMB‑L, the Bplus can highlight abilities prone to a high risk for loss of independence and thus facilitate the prevention of progressive care dependency.

## Operationalisierung der Bestimmung des Barthel-Index mittels „Barthel plus“

Der „Barthel plus“ (Bplus) stellt eine vom Hamburger Einstufungsmanual zum Barthel-Index in seiner Langfassung (HEMB-L) ausgehende Operationalisierung des Barthel-Index dar, die weitere Definitionslücken schließt und eine einfachere Sprache verwendet. Bei voller Punktzahl auf Item-Ebene soll vermerkt werden, ob die Ausführung dennoch beeinträchtigt war (b) oder nicht (a).

### Hintergrund und Zielsetzung

Der Barthel-Index [1] ist das in Deutschland geläufigste Instrument zur Erhebung der Selbstständigkeit bei täglichen Basisaktivitäten und das einzige der vier vom European Network for Action on Ageing and Physical Activity (EUNAAPA) empfohlenen [2], das in der S1-Leitlinie „Geriatrisches Assessment der Stufe 2, Living Guideline“ [3] genannt wird. Durch das „Hamburger Einstufungsmanual“ verbesserten Lübke et al. [4] die Standardisierung. Nur die Langfassung (HEMB-L) ist validiert. Der Vergleich der Summen-Scores bei je über 2600 geriatrischen Patienten, die vor vs. nach Einführung des HEMB‑L behandelt worden waren, zeigte keine signifikante Differenz trotz stärkerer Verlagerung des Fokus vom Funktionsstatus auf den Pflegeaufwand [5], z. B. Punktgewinn durch Vorhandensein von Ernährungssonde oder Urinkatheter.

Die Verwendung von Operationalisierungen, die zu einem vom HEMB‑L abweichenden Summen-Score führen, ist zurzeit in Deutschland abrechnungsrelevant (OPS 8‑550) [6]. Die Verständlichkeit des HEMB‑L ist jedoch für Personen ohne hohe Sprachkompetenz erschwert; auch entspricht die Wortwahl nicht durchgängig aktuell üblichen Formulierungen.

In der Absicht, die Fehlerquote bei der Erhebung des Barthel-Index durch einfachere Sprache zu verringern, weitere Definitionslücken zu schließen und eine Möglichkeit zu schaffen, Beeinträchtigungen unterhalb des Verlusts von Selbstständigkeit punktneutral zu dokumentieren, erfolgte in der Forschungsgruppe Geriatrie Lübeck ab 2015 die Entwicklung einer Operationalisierung, die – unter Berücksichtigung des amerikanischen Originals – auf dem HEMB‑L aufbauen und einen identischen Summen-Score generieren sollte. Dieser „Barthel plus“ (Bplus) wurde erstmals 2016 vorgestellt [7], seine finale Form und Validierung in diesem Artikel.

### Barthel plus

**Abb. 1:**
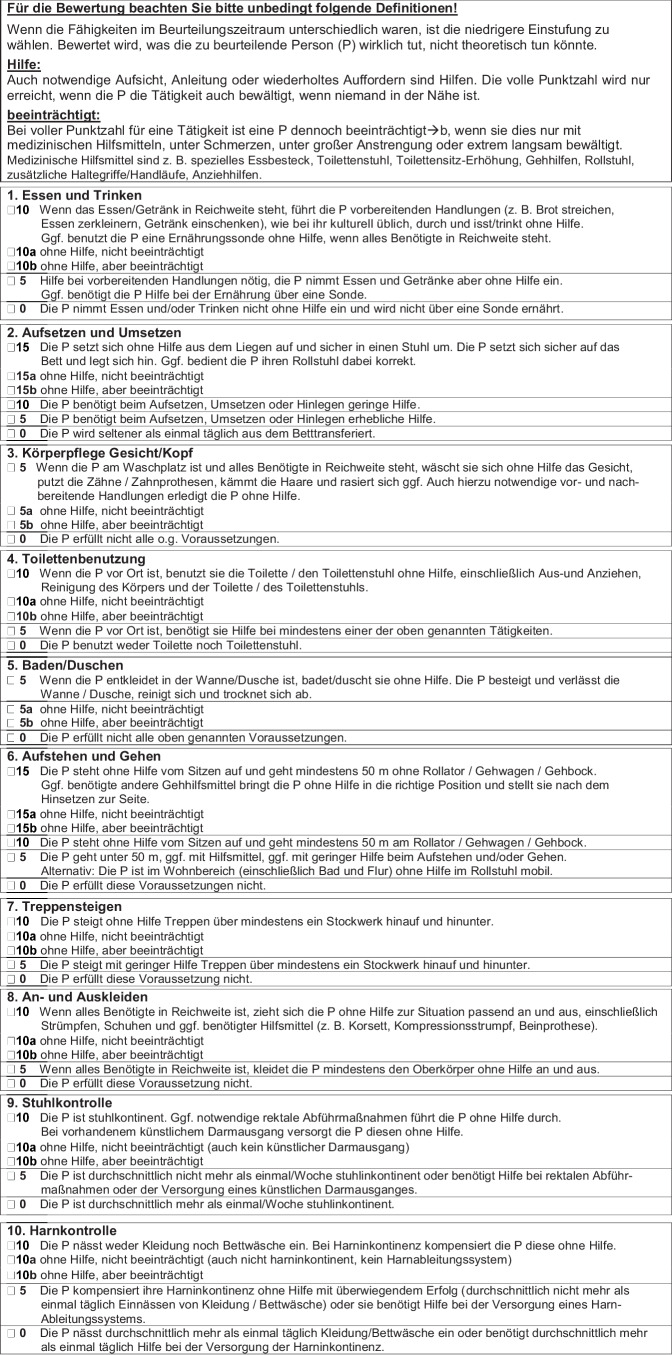
Einstufungsmanual zur Erhebung des Barthel-Index gemäß Barthel plus

Bei der Entwicklung des Bplus wurden Verständnisschwierigkeiten des Pflegefachpersonals bei der Anwendung des HEMB‑L analysiert und in mehreren Feedback-Runden nach einfacheren, eindeutigen Formulierungen gesucht. Hinweise auf Definitionslücken wurden aufgegriffen und versucht, diese zu schließen. Der finale Text weist in der Anleitung mit einem Lesbarkeitsindex [8] von 46,21 (HEMB-L: 59,59) einen mittleren, im Text zu den Items mit 38,56 (HEMB-L: 55,48) einen niedrigen Komplexitätsgrad auf (HEMB-L: hohe Komplexität). Bei Bewertung eines Items mit voller Punktzahl soll das Fehlen bzw. Vorhandensein einer kompensierten Beeinträchtigung durch den Zusatz „a“ bzw. „b“ gekennzeichnet werden – in der Regel bereits bei der ersten Erhebung, spätestens aber bei Entlassung aus geriatrischer Behandlung. Abb. 1 stellt das Instrument vor, Tab. 1 erläutert einige der Umformulierungen. In einer Pilotstudie auf zwei Stationen des Krankenhaus XXX zeigte sich keine signifikante Abweichung der Summen-Scores gemäß Bplus und HEMB‑L. Besonders in der Tagesklinik wurde der Informationsgewinn durch die Subskalierung bei erhaltener Selbstständigkeit als wichtig beurteilt, um Therapiebedarf zu dokumentieren.

**Tab. 1:** Erklärungen zu einigen Formulierungsunterschieden zwischen HEMB‑L (*kursiv*) und Bplus

Allgemein	„*Patient*“ → „Person“: auch für nichtärztliche Perspektive, geschlechtsneutral
	*„Laienhilfe“* → „geringe Hilfe“ (s. *Item 2*)
Item 1	„*Essen*“ → „Essen und Trinken“. Einschenken des Getränks erwähnt
	Statt „*Er nutzt sachgerecht sein Besteck, streicht sein Brot …“ *kultursensible Formulierung
Item 2	*„… wird … nicht aus dem Bett transferiert“* → „… seltener als einmal täglich …“
Item 3	*„Sich waschen“* → „Körperpflege Gesicht/Kopf“: passt besser zu Zahn(prothesen)pflege, Kämmen und Rasieren; vermindert Risiko der Fehlbewertung, wenn andere Körperteile nicht gewaschen werden können
Item 5	Für 5 Punkte werden mehrere Tätigkeiten gefordert, für 0 Punkte formuliert *„erfüllt diese Voraussetzung nicht“* → präzisiert zu „… erfüllt nicht alle oben genannten Voraussetzungen“
Item 6	10 Punkte: außer *„Gehwagen“ *auch Rollator und Gehbock genannt
	5 Punkte: *„Strecken im Wohnbereich“* → „unter 50 m“*, „am Gehwagen“* → „ggf. mit Hilfsmittel“ (egal, mit welchem oder ohne: bei unter 50 m gibt es keine 10 Punkte)
Item 8	Verzicht auf Begriff *„angepasste Kleidung“*, den viele nicht definieren konnten. Ergänzung um „zur Situation passend“, da falsche Kleidungsauswahl Pflegebedarf verursacht
Item 10	0 Punkte: *„durchschnittlich mehr als einmal/Tag harninkontinent“* ersetzt, da erfolgreiche Kompensation nicht zu Punktverlust führt

### Studiendesign und Untersuchungsmethoden

Für die prospektive diagnostische Kohortenstudie wurden stationäre Patienten im Krankenhaus Rotes Kreuz Lübeck Geriatriezentrum in chronologischer Reihenfolge nach Aufnahmedatum auf das Vorhandensein der Einschlusskriterien überprüft. Ausreichend belastungs- und kommunikationsfähige, nichtisolierte Patienten ohne Hinweis auf kognitive Störung wurden um schriftliche Studieneinwilligung gebeten. Die Untersucherin führte die Basisbefragung in der ersten Behandlungswoche (t0) durch. Diese schloss neben dem Bplus auch die Erhebung der Lebensqualität über die Abfrage der 8 fünfstufig selbstbewerteten Items des EUROHIS-QOL8 [9] ein. Die erste Kontrolle des Bplus erfolgte über einer Woche später (t1) während der stationären Behandlung, telefonische Erhebungen 2 Wochen bis 2 Monate (t2), 11 bis 13 (t3), 23–25 (t4) und 35 bis 37 Monate (t5) nach Entlassung. Für die Übereinstimmung von Bplus und HEMB‑L und die Interrater-Reliabilität wurden Audioaufzeichnungen der Erhebung des Bplus von verblindeten Personen mittels HEMB‑L oder Bplus beurteilt. Die Datenanalyse erfolgte mit SPSS Version 29. Die Stichproben wurden mit Mittelwert, Standardabweichung und Streuung beschrieben. Mittelwerte wurden bei nicht normal verteilten rangskalierten Daten mittels Mann-Whitney-U-Test verglichen, bei normal verteilten Daten mittels t‑Test. Korrelationen wurden nach Spearman berechnet. Das Signifikanzniveau wurde auf α = 0,05 festgelegt. Für die Änderungssensitivität wurde die Effektstärke gemäß Cohens d ermittelt. Für die prognostische Validität wurde das relative Risiko für Punktverluste auf Item-Ebene bei primär voller Punktzahl mit („b“) vs. ohne („a“) Beeinträchtigung ermittelt.

### Ergebnisse

Zwischen 29.04.2019 und 25.06.2021 wurden 126 Patienten für die Studienteilnahme rekrutiert, von denen zu den 6 Untersuchungszeitpunkten 124 (t0), 84 (t1), 70 (t2), 66 (t3), 56 (t4) sowie 42 (t5) befragt werden konnten. Gründe für den Schwund an Teilnehmenden sind in Tab. 2 aufgeführt. Zu allen Zeitpunkten war fehlende Erreichbarkeit der häufigste Grund für Nichtteilnahme.

**Tab. 2:** Übersicht über Gründe für die Nichtteilnahme rekrutierter Patienten t1 bis t5

	Anzahl geplanter und geführter Interviews (*n*)	Kein Interview erfolgt wegen (+ nicht erreicht aus ungeklärten Gründen) (*n*)
t1	124 → 84	12 entlassen, 7 verlegt, 7 in Quarantäne (+14)
t2	124 → 70	10 keine gültige Rufnummer, 3 Abbruch der Studienteilnahme, 2 Gespräch aktuell abgelehnt, 2 aktuell nicht kommunikationsfähig, 2 im Krankenhaus, 1 verstorben (+34)
t3	110 → 66	11 keine gültige Rufnummer, 3 Gespräch aktuell abgelehnt, 1 aktuell nicht kommunikationsfähig, 9 verstorben (+20)
t4	90 → 56	12 keine gültige Rufnummer, 1 Gespräch aktuell abgelehnt, 2 dauerhaft nicht kommunikationsfähig (1 Verlust des Gehörs, 1 schwere kognitive Störung), 1 in Pflegeeinrichtung, 7 verstorben (+11)
t5	69 → 42	7 keine gültige Rufnummer, 2 Abbruch der Studienteilnahme, 1 Gespräch aktuell abgelehnt, 2 dauerhaft nicht kommunikationsfähig bei schwerer kognitiver Störung, 3 in Pflegeeinrichtung, 3 verstorben (+7)

Zu t0 lag das Alter der Patienten bei 81,1 ± 7,3 (60 bis 101) Jahren, Frauen stellten 66,9 % (*n* = 83), Männer 33,1 % (*n* = 41). Der Summen-Score des EUROHIS-QOL8 lag bei den 124 Patienten mit 27,8 ± 4,0 (17 bis 37) Punkten im mittleren Bereich und korrelierte auf mittlerem Niveau (r = 0,336, *p* < 0,001) mit dem Bplus. Dabei korrelierte das Item „Wie zufrieden sind Sie mit Ihrer Leistung bei Alltagsverrichtungen?“ am höchsten (r = 0,472, *p* < 0,001).

Das Intervall t0/t1 betrug 13,6 ± 2,5 (6 bis 21) Tage. Zu t0 lag der Barthel-Index nach Bplus bei 62,1 ± 17,4 (10–100), zu t1 bei 71,9 ± 16,6 (25–100).

Die Summen-Scores von Bplus und HEMB‑L unterschieden sich weder zu t0 noch zu t1 (*p* = 0,199; *p* = 0,612), die entsprechenden Korrelationen lagen bei 0,939 bzw. 0,938 (jeweils *p* < 0,001). Die Interrater-Reliabilität des Bplus war mit einem Intraklassen-Koeffizienten von 0,972 (95 %-Konfidenzintervall 0,958–0,982, *p* = 0,001) hoch (t0, *n* = 93).

Die Items „Essen“ und „Aufsetzen und Umsetzen“ zeigten bessere Werte für Männer, die Summen-Scores wiesen keinen Einfluss des Geschlechts auf. Die interne Konsistenz des Bplus war hoch mit Cronbachs α = 0,984. Von t0 zu t1 stieg der Barthel-Index nach Bplus um 10,7 ± 11,6 Punkte, die Änderungssensitivität gemäß Effektstärke betrug Cohens d = 0,919 (95 %-Konfidenzintervall 0,661–1,172).

Bei 99 Patienten (79,8 %) zu t0 und 74 (88,1 %) zu t1 wurde mindestens ein Item mit voller Punktzahl als beeinträchtigt (b) bewertet. Tab. 3 zeigt für alle Zeitpunkte die Häufigkeit, mit der die Einstufung „b“ gewählt wurde.

**Tab. 3:** Vergabe des vollen Punktwertes auf Item-Ebene und der Bewertung *b*

Item	Punkte	t0 (*n* = 124) (%)	t1 (*n* = 84) (%)	t2 (*n* = 70) (%)	t3 (*n* = 66) (%)	t4 (*n* = 56) (%)	t5 (*n* = 42) (%)
1	10	80 (65)	49 (58)	63 (90)	56 (85)	47 (85)	34 (81)
	10b	28 (23)	19 (23)	7 (10)	13 (23)	10 (18)	12 (29)
2	15	78 (63)	68 (81)	62 (89)	60 (91)	52 (95)	37 (88)
	15b	49 (40)	38 (45)	16 (23)	25 (38)	18 (33)	12 (29)
3	5	111 (90)	81 (96)	68 (99)	59 (89)	49 (89)	37 (88)
	5b	33 (27)	30 (36)	11 (16)	19 (29)	13 (24)	5 (12)
4	5	74 (60)	62 (74)	64 (94)	59 (89)	52 (93)	35 (83)
	5b	36 (29)	28 (33)	19 (28)	31 (47)	22 (39)	14 (33)
5	10	7 (6)	12 (14)	31 (46)	30 (45)	29 (52)	22 (52)
	10b	5 (4)	8 (10)	14 (21)	16 (24)	8 (14)	8 (19)
6	5	13 (10)	21 (25)	35 (51)	29 (44)	25 (45)	19 (45)
	5b	9 (7)	13 (15)	15 (22)	13 (18)	12 (21)	8 (19)
7	15	3 (2)	8 (10)	35 (51)	35 (53)	32 (58)	26 (62)
	15b	2 (2)	6 (7)	14 (21)	17 (26)	12 (22)	14 (33)
8	10	38 (31)	42 (50)	48 (71)	43 (65)	38 (69)	31 (74)
	10b	19 (15)	23 (27)	7 (10)	15 (23)	14 (25)	12 (29)
9	10	102 (82)	73 (87)	63 (93)	59 (89)	48 (87)	32 (76)
	10b	33 (27)	19 (23)	2 (3)	4 (6)	3 (5)	2 (5)
10	10	76 (61)	63 (75)	52 (76)	57 (86)	48 (87)	32 (76)
	10b	24 (19)	20 (24)	12 (18)	28 (42)	29 (53)	16 (38)

Bezüglich mit „b“ vs. „a“ bewerteter Items trat im Verlauf deutlich häufiger ein Punktverlust ein. Das relative Risiko, eine den Barthel-Index tangierende Verschlechterung der Selbstversorgungsfähigkeit zu erleiden, lag für das Intervall t0/t1 bei 1,66, für t1/t2 bei 2,77, für t2/t5 bei 1,53 und für t1/t5 bei 1,90.

## Diskussion

Eine Schwäche des Barthel-Index besteht darin, Interventionsbedarf mit dem Ziel des *Erhalts* der selbstständigen Ausführung der 10 erfassten Alltagstätigkeiten nicht zu erfassen. Modifikationen, die zu einem anderen Summen-Score führen [10, 11], zerstören jedoch seinen größten Vorteil, die internationale Vergleichbarkeit von Daten, und konnten sich nicht durchsetzen. Der Weg, Verbesserungen der Aussagefähigkeit unter Wahrung des Summen-Scores zu erzielen, ist 2001 mit dem HEMB‑L eingeschlagen worden, darf jedoch nicht in eine Sackgasse führen. Schon der kontinuierliche Wandel des Sprachgebrauchs führt inzwischen zu Anpassungsbedarf. Diesem wurde im Zuge der Entwicklung der Operationalisierung nach Barthel plus Rechnung getragen. Die Einführung einer punktneutralen Subskalierung bei Erreichen des Maximalwertes auf Item-Ebene bildet Änderungen des Funktionsstatus zwischen Normalität und Kompensation ab und hat ambulant den Vorteil der Erfassung von Beeinträchtigungen ohne bereits eingetretenen Pflegebedarf. Bei (teil-)stationärer Aufnahme lenkt sie das Augenmerk auf Domänen der Selbstversorgungsfähigkeit, die es – über den offensichtlichen Pflegebedarf hinaus – zu stabilisieren gilt (b) und kennzeichnet die stabilen Ressourcen (a). Ein Vorteil der Nutzung des Bplus für eine prognostische Funktion liegt gegenüber dem Einsatz zusätzlicher Instrumente [12], die global auf ein erhöhtes Risiko für zunehmende Pflegebedürftigkeit hinweisen, somit darin, spezifisch die Alltagstätigkeiten zu markieren, auf die sich präventive Maßnahmen – neben denen mit bereits eingetretenem Hilfebedarf – richten sollten, da dort ein deutlich erhöhtes Risiko für den Verlust der Selbstständigkeit besteht. Die fast 3‑mal so hohe Rate an punktrelevanten Verschlechterungen auf Item-Ebene innerhalb von 2 Monaten nach Entlassung aus geriatrischer Krankenhausbehandlung bei Selbstständigkeit mit Beeinträchtigung (b) gegenüber ohne diese (a) kann als Auftrag aufgefasst werden, auch kompensierte Beeinträchtigungen zu dokumentieren und zu kommunizieren. Dafür reicht der Summen-Score des Barthel-Index nicht aus. Das Kompetenz-Centrum Geriatrie formulierte bereits vor Jahren: „Zur fachlichen Beurteilung ist mindestens eine Übermittlung der Einzelitemergebnisse erforderlich“ [13]; nur dann kommen die Vorteile des Bplus voll zum Tragen. Es ist zu hoffen, dass die komplette Weitergabe der Ergebnisse der Erhebung aller 10 Items in Zeiten der papierlosen Datenübermittlung weniger als Hindernis gesehen werden wird.

## Fazit für die Praxis

- Die Operationalisierung des Barthel-Index nach Barthel plus verwendet eine einfachere Sprache und schließt weitere Interpretationslücken. Der Summen-Score entspricht dem gemäß dem Hamburger Einstufungsmanual.
- Interrater-Reliabilität, interne Konsistenz und Änderungssensitivität des Barthel plus sind hoch.
- Die Option einer punktneutralen Subskalierung bei höchstem Punktwert auf Item-Ebene zeigt auf, wo ein erhöhtes Risiko für einen Verlust von Selbstständigkeit besteht. Veränderungen innerhalb des Bereichs der selbstständigen Ausführung werden erst durch die Subskalierung dokumentierbar.

## Data Availability

Die erhobenen Datensätze können auf begründete Anfrage in anonymisierter Form beim korrespondierenden Autor angefordert werden. Die Daten befinden sich auf einem Datenspeicher am Krankenhaus Rotes Kreuz Lübeck Geriatriezentrum.
